# *Bauhinia forficata* Link Infusions: Chemical and Bioactivity of Volatile and Non-Volatile Fractions

**DOI:** 10.3390/molecules27175415

**Published:** 2022-08-24

**Authors:** Eliane Przytyk Jung, Beatriz Pereira de Freitas, Claudete Norie Kunigami, Davyson de Lima Moreira, Natália Guimarães de Figueiredo, Leilson de Oliveira Ribeiro, Ricardo Felipe Alves Moreira

**Affiliations:** 1 Laboratory of Organic and Inorganic Chemical Analysis, National Institute of Technology, Rio de Janeiro 20081-312, Brazil; 2 Faculty of Chemical Engineering, Federal University of Fluminense, Niterói 24210-240, Brazil; 3 Laboratory of Natural Products, Rio de Janeiro Botanical Garden Research Institute, Rio de Janeiro 22460-030, Brazil; 4 Laboratory of Tobacco and Derivatives, National Institute of Technology, Rio de Janeiro 20081-312, Brazil; 5 Food and Nutrition Graduate Program, Federal University of Rio de Janeiro State (UNIRIO), Rio de Janeiro 22290-250, Brazil

**Keywords:** “pata-de-vaca”, phytochemical profile, bioactive compounds, antioxidant capacity, α-amylase inhibition, SPME technique

## Abstract

This study aimed to evaluate *Bauhinia forficata* infusions prepared using samples available in Rio de Janeiro, Brazil. As such, infusions at 5% (*w*/*v*) of different brands and batches commercialized in the city (CS1, CS2, CS3, and CS4) and samples of plant material botanically identified (BS) were evaluated to determine their total phenolic and flavonoid contents (TPC and TFC), antioxidant capacity (ABTS^•+^, DPPH^•^, and FRAP assays), phytochemical profile, volatile compounds, and inhibitory effects against the α-amylase enzyme. The results showed that infusions prepared using BS samples had lower TPC, TFC and antioxidant potential than the commercial samples (*p* < 0.05). The batch averages presented high standard deviations mainly for the commercial samples, corroborating sample heterogeneity. Sample volatile fractions were mainly composed of terpenes (40 compounds identified). In the non-volatile fraction, 20 compounds were identified, with emphasis on the CS3 sample, which comprised most of the compounds, mainly flavonoid derivatives. PCA analysis demonstrated more chemical diversity in non-volatile than volatile compounds. The samples also inhibited the α-amylase enzyme (IC_50_ value: 0.235–0.801 mg RE/mL). Despite the differences observed in this work, *B. forficata* is recognized as a source of bioactive compounds that can increase the intake of antioxidant compounds by the population.

## 1. Introduction

*Bauhinia* is a genus comprising over 300 species widely distributed in tropical and subtropical forests. In Brazil, 64 species belonging to the Fabaceae family were identified and are commonly known as “pata-de-vaca” due to the shape of their leaves. Most species are of Asian origin; however, *Bauhinia longifolia* (Bong.) Steud. and *Bauhinia forficata* Link are native species from Brazil [1,2].

*B. forficata* is widely used in Brazilian folk medicine due to its beneficial effects on different diseases and human disorders such as rheumatism, local pain, uric acid, and uterine problems [3], but it is primarily used to treat type II diabetes [4]. The beneficial effects are associated with various biocompounds present in *B. forficata*, such as flavonoids, alkaloids, and terpenes/terpenoids [2,5]. The flavonoid compounds are highlighted since they are the major class in *B. forficata* extracts. Farag et al. [6] registered the presence of quercetin and kaempferol derivatives in different species of the *Bauhinia* genus, including *B. forficata*.

In Brazil, *B. forficata* is mainly commercialized dried and used to prepare infusions. Thus, under Brazilian law, the *Bauhinia* tea is associated to food products, so it is not mandatory to indicate the content of bioactive or toxic compounds, as in a limited manner in herbal products [7]. *B. forficata* infusions were used in different in vivo studies, such as that reported by Salgueiro et al. [8], who evaluated the effects of infusions on oxidative stress, liver damage, and glycemia in mice. Nevertheless, data on the content of bioactive compounds, antioxidant capacity, and volatile compounds, among other parameters of this plant, to compare botanically identified and commercialized samples and their infusions are scarce in the literature. Since it is well known that various factors such as climate, processing, and storage conditions may influence the content of bioactive compounds and the volatile fraction of medicinal plants [9,10], there is a clear need for further studies.

Despite that, to date, there are no data available on the volatile composition of *B. forficata* infusions. This fraction cannot be underestimated since *B. forficata* is prepared by infusion or decoction and, therefore, some of the volatile content may disperse in the beverage (hydrolate) and contribute to its beneficial actions besides the aroma. This approach has already been evaluated for other medicinal plants, and the migration of terpenoid and other compounds classes present in the essential oil of the plant for infusion was observed [11,12].

For such an evaluation, headspace solid-phase microextraction coupled to gas chromatography–mass spectrometry (HS-SPME/GC–MS) has been reported as a fast, sensitive, and solvent-free technique for analyzing the extraction and isolation of volatile and semi-volatile compounds, and it has been widely used since its invention in 1989 [13,14]. Furthermore, interference from the infusion matrix may be drastically reduced while the headspace analytes are trapped in the fiber [15]. Thus, this technique has been successfully applied to analyze volatile compounds in infusions and teas [16,17].

In this sense, this work aimed to perform a comprehensive chemical characterization of the volatile and non-volatile fractions of botanically identified and commercial samples of *B. forficata* used to prepare infusions at 5%. The antioxidant capacity measured by ABTS**^•+^**, DPPH**^•^** and FRAP assays and inhibitory activity of α-amylase of the samples were also determined.

## 2. Results

### 2.1. Bioactive Compounds and Antioxidant Capacity of B. forficata Infusions

The TPC, TFC and antioxidant capacity of the *B. forficata* infusions are summarized in Table 1. It should be pointed out that the results presented in this study for TPC and TFC are expressed as rutin equivalents (RE) since this compound belongs to the flavonoids class, which is the major class in this species [6]. The values of TPC varied from 1923 to 6355 mg RE/100 g. Compared to the literature, the highest value found in this study, which was for the dry basis (7222 mg RE/100 g), is superior to that reported by Port’s et al. [18], who evaluated different infusions of herbs from the Brazilian Amazonian region. Even though these authors did not evaluate *B. forficata*. However, their approach was the closest to this study, reporting results of the chemical evaluation for a *B. ungulata* infusion at 2% (g/mL) (2367 mg GAE/100 g dry basis). By calculation, at 5%, 5918 mg GAE/100 g dry basis would be found. Comparisons with data from the literature are difficult since few studies used the same species, and even when the species were the same, the results were expressed using different chemical standards, as in the example above. Additionally, it is easier to find data on *B. forficata* extracted with organic solvent than with hot water (infusion). Thus, our discussion will be focused on the differences observed among the brands and respective batches evaluated herein.

The values for TPC, TFC, and antioxidant capacity measured by DPPH^•^, ABTS^•+^, and FRAP assays varied from 1923 to 6355 mg RE/100 g, 482 to 3700 mg RE/100 g, 19 to 206 µmol Trolox/g, 27 to 204 µmol Trolox/g, and 85 to 644 µmol Fe^2+^/g, respectively. This corroborates that variations among samples and batches were high (Table 1). Among batches of the commercial samples, the highest values for TPC, TFC and antioxidant capacity (CS4B2 and CS3B3) were observed. These were higher than values reported to botanically identified sample (BS), which may be explained by differences in cultivation practices and the way the plants were processed. For example, the drying time may increase the degradation of plant bioactive compounds, whereas soil characteristics and precipitation conditions may affect the biosynthesis of secondary metabolites [9,10].

CS4B2 presented the highest TPC and FRAP values. For the TFC and DPPH**^•^** and ABTS**^•+^** assays, CS3B3 presented the highest values (Table 1). The literature points to a direct relationship between TPC and antioxidant capacity; however, in this study, the sample that presented the highest TPC did not show the highest values for antioxidant capacity measured by all assays employed. This corroborates that the phytochemical composition of plant extracts may interact differently with radical species, which helps explain the results found.

High standard deviations were observed in CS2 and CS4 samples. The variation coefficient for the TFC reached 75% in CS2, for example, confirming the heterogeneity among the sample batches. The low standard deviation of the BS may be associated mainly with the standardization of the processing, which was followed from the harvest of leaves to drying. In addition, the harvest was from the same tree, although it took place in different seasons. This may also justify the low standard deviation of CS1 and CS3. Furthermore, conditions such as storage time, temperature, and kind of package have influence on the stability of bioactive compounds.

Since the samples showed heterogeneous batches according to the statistical analysis for this set of experiments, two groups were observed from their averages: one composed of the BS, CS1, CS2, and CS4 groups, for which no statistically significant differences were observed for the TPC, TFC, DPPH**^•^**, ABTS**^•+^**, and FRAP assays (*p* > 0.05), and the other represented by CS3 alone. These data provide important information about the production chain of *B. forficata*, rendering evident the need to standardize the steps that involve from harvest to distribution to deliver to consumers a product that guarantees its bioactive properties. *B. forficata* is widely used in Brazilian folk medicine due to its beneficial effects for treating rheumatism, local pain, uric acid, uterine problems [3], and, especially, type II diabetes [4]. This is possible due to the phytochemical profile of *B. forficata*, which is mainly composed of flavonoids, recognized for their antioxidant capacity [19].

### 2.2. LC-HRMS Analysis

A total of 20 phenolic compounds (Table 2), among flavonoids, phenolic acids, and other phenolic compounds, were tentatively identified in the samples. The majority are kaempferol and quercetin derivatives. The samples comprised flavonoid *O*-glycosides, thus in accordance with previously reported results, which prove its pharmacological action [20,21]. Additionally, polar compounds were identified in the samples in accordance with the polarity of the infusions.

Most phenolic compounds identified in this study were free phenolic compounds, esterified with sugars or other compounds with low molecular masses, such as quercetin 3-*O*-rhamnoside, Kaempferol 3-*O*-glucoside, and Isorhamnetin.

Rutin, Isoquercetin, Quercetin-*O*-pentoside, Quercetin 3-*O*-rhamnoside, Kaempferol 3-*O*-glucoside, Kaempferol 3-*O*-rutinoside and Isorhamnetin were the compounds detected in all samples. CS3 is the infusion with the greatest number of compounds that vary according to the batch. In CS1, CS2, and CS4, the same 11 flavonoids were identified with differences in the relative abundance of the ions. Compound 3 showed a precursor ion [M–H]¯ at 331.0670 *m*/*z* and a typical loss of a hexose in MS^2^ resulting in a [M–H]¯ *m*/*z* 169 fragment. It was assigned as galloyl hexose. Compound 4 was assigned as hydroxibenzoic acid based on precursor ion [M–H]¯ at 137.0244 *m*/*z* and a very low error between experimental and theoretical mass of 0.1 ppm [22]. Compound 6 showed a precursor ion [M–H]¯ at 353.0875 *m*/*z* and the quinic acid fragment in MS^2^ at *m*/*z* 191, been identified as 3-Caffeoyl quinic acid. Compound 10 was assigned as Quercetin 3-*O*-glucopyranoside by comparison with literature records (1 ppm error ([M–H]¯ *m*/*z* 463.0878) [23]. Compound 19 was assigned as Isorhamnetin 3-*O*-rhamnosyl-rutinoside based on precursor ion [M–H]¯ *m*/*z* 769.2190 and based in the loss of Isorhamnetin fragment at *m*/*z* 315. Kaempferol fragment ion at *m*/*z* 284 was used to identify compound 20 as Kaempferol 3-*O*-dirhamnoside along with the precursor ion [M–H]¯ *m*/*z* 577.1595 [6]. Identification of the other listed compounds by fragmentation data and exact mass were previously described by the authors [24,25].

The UPLC-ESI-Q-TOF MS/MS chromatographic technique was an efficient tool to characterize and identify the phenolic compounds in *B. forficata* infusions. It is important to highlight that the advantage of this technique is that, although it is not quantitative, one may relatively quantify the compounds, even the isomeric forms (e.g., Catechin, *Epi*-catechin, and Quercetin-*O*-pentoside), and, in case of a lack of standards, the compound assignments may be made by comparison of UV spectra and MS data (accurate mass and fragmentation) with previous literature reports [6,22,23].

**Figure 1:**
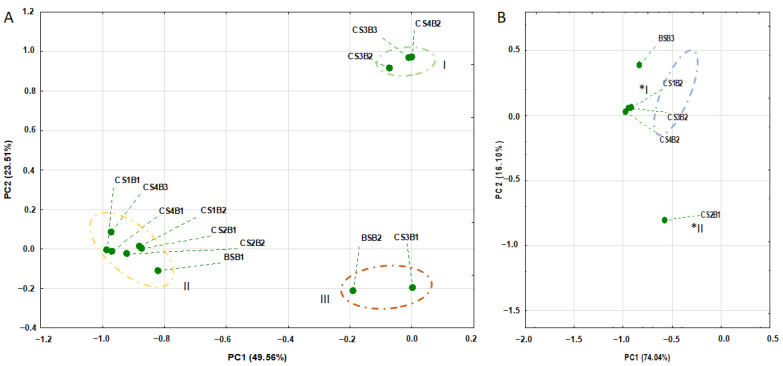
Principal component analysis of (**A**) non-volatile compounds and (**B**) volatile compounds. BS: botanic sample; CS1: commercial sample 1; CS2: commercial sample 2; CS3: commercial sample 3; CS4: commercial sample 4. B is relative to the batch. *I—BSB3 (rich in Caryophyllene oxide), CS1B2, CS3B2 and CS4B2 (rich in Spathulenol); *II—CS2B1 (rich in 2-Propyl-1-heptanol).

A PCA analysis of the non-volatile chemical composition showed three distinct groups: **I**—CS3B2, CS3B3 and CS4B2; **II**—CS1B1, CS1B2, CS2B1, CS2B2, CS4B1, CS4B3 and BSB1; **III**—CS3B1, BSB2 (Figure 1A). These results demonstrate great chemical variability between the different samples, although flavonoids Rutin, Isoquercetin, Quercetin-*O*-pentoside, Quercetin 3-*O*-rhamnoside, Kaempferol 3-*O*-glucoside, Kaempferol 3-*O*-rutinoside and Isorhamnetin were detected in all samples.

### 2.3. HS-SPME/CG–MS

The identification and relative concentrations of the volatile compounds in the five herbal infusions of *B. forficata* are shown in Table 3, in order of retention time (Rt), and increasing Linear Retention Index (LRI). Forty volatile compounds were tentatively identified, of which only seven were detected in all samples: 2-Propyl-heptanol (7.69–19.42%), Geranyl acetone (4.38–7.31%), Dodecanol (3.11–11.37%), β-Ionone (0.71–5.84%), Spathulenol (11.78–30.87%), Caryophyllene oxide (2.76–17.46%), and Benzoic acid 2-ethylhexyl ester (1.12–20.14%). The volatile compounds included terpenoids, represented by C_13_-norisoprenoids, sesquiterpenes, and monoterpenes, as well as hydrocarbons, alcohols, esters, aldehydes, ketones, and acids. Among all chemical groups found in the volatiles of the *B. forficata* infusions, sesquiterpenes (hydrocarbon and oxygenated) were present in a higher number (17) and represented most of the composition of the BS (63%), CS1 (61%), CS3 (50%), and CS4 (53%). Esters (31%) and alcohols (29%) accounted for most of the composition of CS2.

There are no data in the literature on the volatile composition of *B. forficata* infusions or any species of the *Bauhinia* genus. However, there are two studies that identified constituents of essential oils of this species and demonstrated that they are essentially composed of sesquiterpenoids. Duarte-Almeida et al. [26] and Sartorilli and Correa [27] evaluated the composition of essential oils in *B. forficata* and reported that the content of sesquiterpenoids was 87% and 96%, respectively. Our results and those from essential oils [26,27] are a great evidence that a mostly sesquiterpenic volatile fraction composition may be characteristic of this species.

It is well established that many sesquiterpenes and their alcohol, aldehyde, and ketone derivatives are biologically active or precursors of metabolites with biological functions, while others have desirable fragrance and flavoring properties [28]. Spathulenol (8.53–25.86%) and Caryophyllene oxide (2.76–17.46%) were two of the major compounds in all samples. Both compounds are known to possess several biological activities. Nascimento et al. [29] demonstrated antioxidant, anti-inflammatory, antiproliferative, and antimycobacterial activities of spathulenol, and a moldy and herbaceous odor is attributed to this compound [30]. In turn, Caryophyllene oxide has a floral and woody odor [31,32], and biological activities such as anticholinesterase, analgesic, anti-inflammatory, and antifungal activities were also reported [33,34]. Regarding the class of norisoprenoids (C_13_), they were detected in all samples at concentrations ranging from 7.56% to 14.71%, highlighting Geranyl acetone and β-Ionone. It is reported that they present a significant aromatic impact in fruits such as grapes, apples, lychee, and mango [35,36], with a floral odor being attributed to them [37].

Attention is drawn to the identification of Bisphenol A (BPA) and Dibutyl phthalate (DBP) in some samples evaluated here, especially CS2, which showed important concentrations of these contaminants in its volatile fraction (9.24% and 3.82%, respectively). As any agricultural product, these herbs may be subjected to chemical contaminations due to agricultural practices, especially in stages when a plastic material is used as packaging or support or due to soil treatment, cultivation in contaminated soil, and other factors [38,39]. Furthermore, the migration of these plasticizers that constitute the packaging cannot be ruled out since it is known that this is the main source of exposure to this type of contaminant [39]. Di Bella et al. [39] and Lo Turco et al. [40] evaluated the BPA contamination of spices and herbs from different origins and found it to be present in several samples. Despite concluding that the ingestion of these contaminants does not imply a risk to human health, one cannot disregard their existence, and mechanisms to mitigate them must be evaluated, such as proposing other packaging materials free from them.

In general, the observed differences among the volatile fraction patterns of the infusions were lower than those observed for non-volatile (Figure 1B). Only CS2B2 formed another group by PCA analysis (Figure 1B). Indeed, different origins of the samples with their unique ecological settings as well as features intrinsic to the medicinal herbs may explain this difference [12]. Moreover, Arsenijević et al. [12] stressed that compounds present in the volatile fraction of infusions play an important role in the antioxidant capacity of these products, thus rendering this evaluation relevant, although it was still not possible to measure it in this work. Once again, we highlight that the results obtained herein are the first step towards revealing the beneficial health effects of *B. forficata* infusions through chemical diversity after evaluating their non-volatile and volatile fractions.

### 2.4. Assay for α-Amylase Inhibition

In this set of experiments the effect of *B. forficata* infusions that presented better results for TPC, TFC and antioxidant capacity was investigated. The results revealed that all infusions inhibited the α-amylase activity. Based on the IC_50_ values, which represent the concentration required to inhibit 50% of the enzyme activity, the CS2B1 sample was the one that showed the greatest potential for enzyme inhibition, as it showed the lowest IC_50_ value (0.235 mg RE/mL). The IC_50_ values were 0.235 mg RE/mL, 0.245 mg RE/mL, 0.287 mg RE/mL, 0.489 mg RE/mL, and 0.801 mg RE/mL for CS2B1, CS4B2, CS1B2, BSB3, and CS3B2, respectively. Even though CS4B2 presented the highest TPC, this sample exhibited a higher IC_50_ value. It is suggested that the inhibition of α-amylase activity may be due to other phytochemicals also present in the infusions such as terpenoids, which were detected in the samples by HS-SPME/CG–MS. However, it is well known that phenolic compounds, mainly flavonoids, are excellent inhibitors of digestive enzymes. Flavonoids and their derivatives have the ability to reduce the potency of α-amylase and α-glucosidase by either interacting with or inhibiting specific positions of the enzyme [41]. However, other classes of compounds should not be neglected as published by Papoutsis et al. [42], which reported in their review the positive effects of terpenoids, carotenoids, among others compounds on inhibition of α-amylase activity. It is important to note that these compounds should be bioavailable after digestion to act on digestive enzymes. Thus, future studies on this subject should be addressed.

Acarbose is widely used in medicine as an inhibitor of digestive enzymes related to the breakout of polysaccharides. As these enzymes are inhibited, there is a reduction in glucose absorption and, consequently, a decrease in the postprandial blood glucose level elevation, which helps reduce the risk of Diabetes mellitus, for example [42]. Its IC_50_ value was found to be 0.034 mg/mL. Thus, a lower concentration of this substance is required to inhibit 50% of the α-amylase activity when compared to *B. forficata* infusions. However, it should be noted that this medicinal plant is widely used in folk medicine as an adjuvant in treating hyperglycemia by the population, especially those in vulnerable conditions [43].

It is important to demonstrate that infusions prepared from commercially available herbs showed an important inhibitory action on the enzyme despite being less potent than acarbose. Furthermore, cytotoxicity was not observed when different fractions from *B. forficata* were evaluated by Franco et al. [44]. These facts reinforce the biological and pharmacological potential of *B. forficata* as hypoglycemiant agent, which has an important role in Brazilian folk medicine, primarily because it is abundant and easily accessible.

## 3. Material and Methods

### 3.1. Plant Material

*B. forficata* leaves were collected in Petropolis, Rio de Janeiro, Brazil (22°30′04.63″ S, 43°07″58.20″ W, altitude: 958 m) in different seasons (winter, spring, and summer-2018/2019). Voucher specimens were deposited at the Herbarium of the Department of Botany of the Federal University of Rio de Janeiro, under registration number RFA 40.615. The samples were dried in an oven with forced air circulation at 45 °C, then disintegrated in a domestic blender to obtain a powered material, which was used to prepare the infusions. These samples were named BSB1 (winter), BSB2 (spring), and BSB3 (summer).

Four commercial samples purchased from local markets in the city of Rio de Janeiro were also evaluated. Two batches of commercial sample 1 (CS1) and three batches of the other samples (CS2, CS3, and CS4) were acquired, resulting in samples CS1B1, CS1B2, CS2B1, CS2B2, CS2B3, CS3B1, CS3B2, CS3B3, CS4B1, CS4B2, and CS4B3, which were used to prepare the infusions.

### 3.2. Preparing the Infusions

The infusions were prepared by adding 50 mL of boiling water to 2.5 g of the samples (5% *w*/*v*). After that, they were allowed rest at room temperature for 20 min. The extracts were filtered and transferred to a volumetric flask, in which the volume was quenched with distilled water until reaching 50 mL [45].

### 3.3. Analysis

#### 3.3.1. Total Phenolic Content (TPC)

The TPC analysis was performed using the Folin-Ciocalteu reagent (Imbralab, Ribeirão Preto, Brazil), following the method described by Singleton and Rossi [46]. For the reactions, 250 μL of the filtered and appropriately diluted extract was mixed with 1250 μL of 10% Folin-Ciocalteu reagent and 1000 μL of a 7.5% (*w*/*v*) sodium carbonate solution. Thereafter, the samples were heated at 50 °C for 15 min and cooled at room temperature. The absorbance was measured at 760 nm. A calibration curve was constructed using the rutin (Sigma-Aldrich, St Louis, MO, USA) standard with concentrations ranging from 16 mg/L to 166 mg/ L (linear regression: y = 0.0034x−0.0128; R^2^ = 0.9988). The TPC is expressed as milligrams of rutin equivalent per 100 g (mg RE/100 g).

#### 3.3.2. Total Flavonoid Content (TFC)

The TFC was determined based on the method described by Zhishen et al. [47] with minor modifications. Here, 0.5 mL of extract was mixed with 3.2 mL of ultrapure water and 150 μL of NaNO_2_ (5%, *w*/*v*). After homogenization, the mixture was left to rest for 5 min. Thereafter, 150 μL of AlCl_3_ (10%, *w*/*v*) was added to the mixture, and 1 mL of NaOH (1 M) was added after 1 min. The absorbance was recorded at 510 nm with a spectrophotometer (Metash, Shanghai, China) using ultrapure water as a blank. The TFC was calculated using the calibration curve of rutin (Sigma-Aldrich, St. Louis, MO, USA) standard, with the concentration ranging from 99 mg/L to 595 mg/L (linear regression: y = 0.001x + 0.013; R^2^ = 0.9974). The results are expressed as mg RE/100 g.

#### 3.3.3. ABTS^•+^ Assay

The antioxidant capacity was determined by the reduction of radical monocation, 2,2′-azinobis-(3-ethylbenzothiazoline-6-sulfonic acid) (ABTS^•+^), according to the procedure described by Gião et al. [48]. The radical was obtained after the addition of 7 mmol/L of ABTS (2,2′-azinobis-(3-ethylbenzothiazoline-6-sulfonic acid) diammonium salt (Sigma-Aldrich, Saint Louis, MO, USA) to 2.45 mmol/L of a potassium persulfate solution (1:1 (*v*/*v*)). The mixture was left to react in the dark for 16 h. To obtain an absorbance of 0.700 ± 0.020 at 734 nm, the ABTS^•+^ solution was diluted using ultrapure water. For the reactions, 30 μL of each filtered and diluted extract was mixed with 3000 μL of the ABTS^•+^ solution. After 6 min, the absorbance was measured at 734 nm with a spectrophotometer (Metash, Shanghai, China) using ultrapure water as a blank. The ABTS^•+^ antiradical activity was calculated using Trolox solutions (Sigma-Aldrich, Buchs, Switzerland) with different concentrations ranging from 240 to 2000 μmol (linear regression: y = 0.0003x + 0.0094; R^2^ = 0.9989). The results are expressed as μmol of Trolox equivalents per gram (μmol TE/g).

#### 3.3.4. DPPH^•^ Assay

The 2,2′-diphenyl-β-picrylhydrazyl radical (DPPH^•^) (Sigma-Aldrich, Steinheim, Germany) scavenging activity of the extracts was determined according to the method described by Hidalgo et al. [49]. For the reactions, 100 μL of each diluted extract was added to 2900 μL of a DPPH^•^ solution (6 × 10^−5^ M in methanol and diluted to an absorbance of 0.700 at 517 nm). The resulting solutions were allowed to stand for 30 min in the dark at room temperature. Then, the absorbance was measured at 517 nm with a spectrophotometer (Metash, Shanghai, China) using methanol as a blank. The DPPH^•^ scavenging activity was calculated using Trolox solutions (Sigma-Aldrich, Buchs, Switzerland) with different concentrations ranging from 80 to 680 μmol (linear regression: y = 0.0008x + 0.017; R^2^ = 0.9962). The results are expressed as μmol TE/g.

#### 3.3.5. FRAP Assay

The ferric reducing/ antioxidant power (FRAP) assay was performed according to the procedure reported by Benzie and Strain [50] with minor modifications. The stock solutions included 300 mM of an acetate buffer (pH 3.6), 10 mM of 2,4,6-tri(2-pyridyl)-s-triazine (Sigma-Aldrich, Buchs, Switzerland) in 40 mM of HCl, and 20 mM of FeCl_3_⋅6H_2_O. The working solution was prepared by mixing 25 mL of the acetate buffer, 2.5 mL of the TPTZ solution, and 2.5 mL of FeCl_3_⋅6H_2_O. Thereafter, 100 μL of each extract was reacted with 3000 μL of the working solution at 37 °C for 30 min, and the absorbance was measured at 593 nm. The FRAP activity was calculated using FeSO_4_⋅7H_2_O solutions with different concentrations ranging from 150 to 1200 μmol of Fe^2+^ (linear regression: y = 0.0008x + 0.0042; R^2^ = 0.9992). The results are expressed as μmol of Fe^2+^ per gram (μmol Fe^2+^/g).

#### 3.3.6. LC-HRMS Analysis

The sample extract was dissolved in an aqueous solution containing formic acid (0.1%, *v*/*v*) and subjected to an ultra-performance liquid chromatography-quadrupole/time-of-flight mass spectrometry (UPLCqTOF/MS; maXis Impact, Bruker Daltonics, Billerica, MA, USA) analysis. The separation was performed using a Hypersil C18 column (3 μm particle size, 2.1 mm × 150 mm). The column temperature was maintained at 40 °C. Subsequently, an aliquot of 20 μL was injected into the UPLC-ESI-qTOF system with a flow rate of 0.27 mL/min. The linear gradient elution of A (0.1% formic acid in water) and B (acetonitrile) was applied by employing the following method: 5% of B at the beginning; 5% to 9% of B for 5 min, 9% to 16% of B for 10 min, 16% to 36% of B for 18 min, 36% to 95% of B for 1 min, 95% of B for 12 min, 95% to 5% of B for 1 min, and 5% of B for 13 min. Data Analysis 4.2 software (Bruker Daltonics, Billerica, MA, USA) was used to interpret the data. The MS data were acquired in the negative mode using an electrospray ionization (ESI) source. The data were scanned for each test sample at a mass-to-charge ratio (*m*/*z*) from 50 to 1200. Highly pure nitrogen was used as the nebulizing gas and ultrahigh purity helium as the collision gas, and the capillary voltage was set at 5000 V. The ESI parameters included dry gas at 200 °C at a flow rate of 8 L/min and a nebulizer pressure of two bar [25].

#### 3.3.7. HS-SPME/CG–MS

The infusions that presented better results for TPC, TFC and antioxidant capacity were subjected to an analysis of the volatile fraction by Headspace Solid-Phase microextraction followed by gas chromatography–mass spectrometry (HS-SPME/GC–MS).

The headspace volatiles analysis using SPME described by Wang et al. [51] was adopted with minor modifications. Volumes of 10 mL of freshly prepared infusions were placed into 20 mL clear glass vials and immediately capped and placed on a temperature-controlled water bath at 60 °C for 60 min with a SPME fiber coated with 100 µm of PDMS (100% polydimethylsiloxane; Supelco^®^, Bellefonte, PA, USA) pre-conditioned at 250 °C for 60 min and inserted into the headspace above the liquid surface. A system blank with an empty vial was run as a control assay. SPME fibers were desorbed at 250 °C for 5 min in the injection port of the chromatographic system described below.

The GC–MS analysis of the volatile fractions was carried out using an Agilent 6890N gas chromatograph (Agilent Technologies, Palo Alto, CA, USA) with an HP-5MS 5% phenylmethylsiloxane capillary column (30 m × 0.25 mm, 0.25 µm film thickness; Restek, Bellefonte, PA, USA) equipped with an Agilent 5975 mass selective detector in the electron impact mode (ionization energy: 70 eV) operating according to the following conditions. The oven temperature was initially maintained at 60 °C for one 1 min, then raised at the rate of 8 °C/min to 300 °C, staying at this temperature for 15 min. The injector and detector temperatures were set at 250 °C and 260 °C, respectively. The samples were injected in the splitless mode. A normalization technique was used to obtain quantitative data. Linear retention indices (LRI) were calculated for all components using a homologous series of *n*-alkanes (C7–C30, Sigma-Aldrich, Laramie, WY, USA) analyzed under the same conditions as the samples. The identification of the volatile fraction components was based on LRI relative to *n*-alkanes and computer matching with the Wiley275.L and Wiley7n.L libraries and comparisons of the fragmentation patterns of the mass spectra with published data [52].

#### 3.3.8. Assay for α-Amylase Inhibition

The infusions that presented better results for TPC, TFC and antioxidant capacity were subjected to the inhibition assay for α-amylase, performed as reported by Meng et al. [53] with minor modifications. Briefly, 100 μL of extract was mixed with an α-amylase solution (100 μL, 1.0 U/mL) (Sigma-Aldrich, St. Louis, MO, USA) in a phosphate buffer (pH 6.9) and 250 μL of a 1% starch solution. The incubation was carried out for 5 min at 37 °C. The enzyme reaction was stopped by adding dinitrosalicylic acid reagent (250 μL) (Sigma-Aldrich, Steinheim, Germany), and incubation was carried out for 15 min in boiling water. For the dilution, 2 mL of distilled water was added to the final reaction mixture. The absorbance was measured at 540 nm. The inhibitory effect was calculated according to Equation (1), where Abs_control-1_ results from the reaction without adding the enzyme, which was replaced by the buffer solution, while the mixture of the enzyme and starch solution without extract was Abs_control-2_. The results were expressed as IC_50_ (mg RE/mL). Acarbose (Supelco, Laramie, WY, USA) was used as a positive control to compare the inhibitory effects.

Inhibition percentage (%) = [1 − (Abs_sample_−Abs_control-1_)/Abs_control-2_] × 100 (1)

### 3.4. Statistical Analysis

The data were statistically analyzed using Statistica software version 13 (Dell Inc., Tulsa, OK, USA), performing an analysis of variance (ANOVA) and Tukey’s test to verify the differences among averages, considering the 95% confidence level. Experiments were performed in duplicate/triplicate, and the results are presented as the average ± standard deviation. Additionally, the principal component analysis (PCA) were used to assess the variance in the non-volatile and volatile samples. Results were processed using STATISTICA software version 10 (StartSoft Inc., Tulsa, OK, USA).

## 4. Conclusions

It is concluded that the samples presented different TPC, TFC and antioxidant potentials. The commercial CS4B2 and CS3B3 samples showed higher values for bioactive compounds and antioxidant capacity than botanically identified samples. However, both were mostly composed of flavonoid derivatives. PCA analysis demonstrated more chemical diversity in non-volatile than volatile compounds. This analysis may justify the differences observed in the results of the performed assays. To the best of our knowledge, this is the first time that volatile fraction obtained from *B. forficata* infusions has been carried out. It is very clear that it is an important fraction with regard to the aroma besides possible contribution to the biological properties. An inhibitory effect of all *B. forficata* infusions on the α-amylase enzyme was observed. Despite the differences reported in this work, *B. forficata* presents itself as a source of bioactive compounds that may increase the intake of antioxidant compounds by the population.

## Figures and Tables

**Table 1:** Total phenolic content (TPC), total flavonoid content (TFC) and antioxidant capacity of *B. forficata* infusions.

Samples	Assays				
	TPC ¹	TFC ¹	DPPH^•^ ²	ABTS^•+^ ²	FRAP ³
BSB1	2126 ± 15 ^g,h^	648 ± 19 ^e^	20 ± 2 ^e,f^	27 ± 4 ^f^	89 ± 3 ^h^
BSB2	2126 ± 29 ^g,h^	630 ± 9 ^e^	19 ± 0 ^f^	30 ± 2 ^e,f^	85 ± 6 ^h^
BSB3	2772 ± 49 ^e^	832 ±11 ^d,e^	21 ± 1 ^e,f^	30 ± 2 ^e,f^	136 ± 3 ^g^
Overall average	2342 ± 324 ^B^	703 ± 97 ^B^	20 ± 1 ^C^	29 ± 3 ^B^	103 ± 25 ^B^
CS1B1	2364 ± 164 ^f,g^	1026 ± 4 ^d^	34 ± 1 ^d,e,f^	41 ± 0 ^d,e,f^	127 ± 2 ^g^
CS1B2	2733 ± 55 ^e,f^	1042 ± 24 ^d^	39 ± 2 ^d,e^	46 ± 2 ^d^	133 ± 4 ^g^
Overall average	2549 ± 230 ^B^	1034 ± 18 ^B^	36 ± 3 ^B,C^	43 ± 3 ^B^	130 ± 4 ^B^
CS2B1	4740 ± 69 ^c^	3122 ± 114 ^b^	108 ± 1 ^c^	99 ± 1 ^c^	242 ± 5 ^e^
CS2B2	2245 ± 79 ^g,h^	944 ± 40 ^d^	37 ± 3 ^d,e,f^	45 ± 2 ^d,e^	120 ± 2 ^g^
CS2B3	3203 ± 215 ^d^	626 ± 30 ^e^	45 ± 2 ^d^	39 ± 1 ^d,e,f^	176 ± 1 ^f^
OverallAverage	3396 ± 1097 ^B^	1564 ± 1178 ^B^	63 ± 34 ^B,C^	61 ± 29 ^B^	179 ± 53 ^B^
CS3B1	4681 ± 251 ^c^	2006 ± 64 ^d^	114 ± 2 ^c^	109 ± 10 ^c^	330 ± 11 ^d^
CS3B2	5448 ± 144 ^b^	2422 ± 147 ^c^	173 ± 3 ^b^	135 ± 12 ^b^	385 ±11 ^c^
CS3B3	4833 ± 166 ^c^	3700 ± 161 ^a^	206 ± 2 ^a^	204 ± 7 ^a^	571 ± 4 ^b^
Overall average	4987 ± 389 ^A^	2710 ± 773 ^A^	164 ± 42 ^A^	149 ± 44 ^A^	429 ± 109 ^A^
CS4B1	2169 ± 89 ^g,h^	1026 ± 18 ^d^	45 ± 2 ^d^	47 ± 3 ^d^	129 ± 1 ^g^
CS4B2	6355 ± 137 ^a^	2628 ± 90 ^c^	185 ± 8 ^b^	149 ± 6 ^b^	644 ± 19 ^a^
CS4B3	1923 ± 4 ^h^	482 ± 15 ^f^	25 ± 1 ^e,f^	27 ± 1 ^f^	86 ± 5 ^h^
Overallaverage	3483 ± 2158 ^A,B^	1378 ± 967 ^B^	85 ± 76 ^B^	74 ± 57 ^B^	286 ± 269 ^A,B^

Abbreviations in the “Samples” column represent the different batches of each one of the brands evaluated. Different lowercase letters in the same column indicate that the results are statistically different (*p* < 0.05). Different uppercase letters in the same column indicate a statistically significant difference among groups (BS, CS1, CS2, CS3 and CS4) (*p* < 0.05). ¹ Results expressed as mg RE/ 100 g. ² Results expressed as µmol Trolox/g. ³ Results expressed as µmol Fe^2+^/g. BSB1 = botanical sample batch 1; BSB2 = botanical sample batch 2; BSB3 = botanical sample batch 3; CS1B1 = commercial sample 1 batch 1; CS1B2=commercial sample 1 batch 2; CS2B1 = commercial sample 2 batch 1; CS2B2 = commercial sample 2 batch 2; CS2B3 = commercial sample 2 batch 3; CS3B1 = commercial sample 3 batch 1; CS3B2 = commercial sample 3 batch 2; CS3B3 = commercial sample 3 batch 3; CS4B1 = commercial sample 4 batch 1; CS4B2 = commercial sample 4 batch 2; CS4B3 = commercial sample 4 batch 3. Results as the mean ± standard deviation (triplicate).

**Table 2:** Tentatively identified compounds of *B. forficata* infusions.

Compounds		*m*/*z* [M–H]^−^ exp.	MS^2^	Molecular Formula [M–H]^−^	Samples				
					BS	CS1	CS2	CS3	CS4
1	Caffeoyl tartarate	311.0401	179; 135	C_13_H_11_O_9_	+				
2	*Epi*-Catechin	289.0718	245; 203	C_15_H_13_O_6_				+	
3	Galloyl hexose	331.0670	169; 125	C_13_H_15_O_10_	+	+	+		+
4	Hydroxibenzoic acid	137.0244	-	C_7_H5O_3_	+	+	+		+
5	Dihydroxibenzoic acid hexoside	315.0719	108; 152	C_13_H_15_O_9_			+	+	
6	3-Caffeoyl quinic acid	353.0875	191	C_16_H_17_O_9_	+	+		+	+
7	Kaempferol 3-*O*-rhamnosyl-rutinoside	739.2136	284	C_33_H_39_O_19_				+	
8	Rutin	609.1468	300	C_27_H_29_O_16_	+	+	+	+	+
9	Myricitrin	463.0880	316	C_21_H_29_O_12_			+		
10	Quercetin 3-*O*-glucopyranoside (Isoquercetin)	463.0917	301; 300	C_21_H_29_O_12_	+	+	+	+	+
11	Quercetin-*O*-pentoside (Quercetin-*O*-arabinoside)	433.0780	300; 301	C_20_H_17_O_11_	+	+	+	+	+
12	Quercetin 3-*O*-rhamnoside	447.0933	284; 285	C_21_H_29_O_11_	+	+	+	+	+
13	Kaempferol 3-*O*-glucoside	447.0975	-	C_21_H_29_O_11_	+	+	+	+	+
14	Kaempferol 3-*O*-rutinoside	593.1533	327; 284; 285	C_27_H_29_O_15_	+	+		+	+
15	Isorhamnetin	315.0502	300	C_16_H_11_O_7_	+	+	+	+	+
16	Isorhamnetin 3-*O*-rutinoside	623.1638	300; 315	C_28_H_31_O_16_	+				
17	Quercetin 3-*O*-rhamnosyl-rutinoside	755.2087	300; 489	C_33_H_39_O_20_	+			+	
18	Isorhamnetin 3-*O*-rhamnosyl-rutinoside	769.2201	605; 315	C_34_H_41_O_20_	+			+	
19	Kaempferol 3-*O*-dirhamnoside	577.1595	431, 285, 284	C_27_H_29_O_14_				+	
20	Kaempferol-*O*-pentoside	417.0833	285, 284, 255, 227	C_20_H_17_O_10_				+	

BS: botanic sample; CS1: commercial sample 1; CS2: commercial sample 2; CS3: commercial sample 3; CS4: commercial sample 4. *m*/*z*—mass to charge ratio; MS^2^—fragments of the second stage of mass spectrometry.

**Table 3:** Tentatively identified compounds of *B. forficata* infusions with their respective relative percentage (%).

Rt (min)	LRI ^(a)^	Compound	Chemical Class	BSB3	CS1B2	CS2B1	CS3B2	CS4B2
14.00	1185	1-Decanal	A	0.10 ± 0.04	-	0.57 ± 0.22	-	-
14.30	1193	2-Propyl-1-heptanol	AL	3.35 ± 0.35	7.69 ± 0.62	19.42 ± 2.52	9.66 ± 4.93	8.96 ± 3.07
16.40	1195	Estragole	PP	-	0.30 ± 0.00	-	-	0.44 ± 0.21
18.44	1357	Eugenol	PP	0.24 ± 0.00	-	-	-	-
20.10	1428	β-Caryophyllene	S	0.85 ± 0.10	-	-	-	-
20.30	1429	α-Ionone	N	3.59 ± 0.47	1.55 ± 0.08	1.64 ± 0.04	-	-
20.90	1448	Geranyl acetone	N	6.88 ± 1.08	7.31 ± 0.00	5.18 ± 0.76	5.02 ± 1.08	4.38 ± 1.25
20.92	1452	α-Humulene	S	1.22 ± 0.45	-	-	-	-
21.00	1461	Alloaromadendrene	S	0.70 ± 0.03	-	-	-	-
21.20	1472	*p*-Benzoquinone	K	-	0.66 ± 0.04	1.50 ± 0.22	0.99 ± 0.03	-
21.40	1480	Dodecanol	AL	4.00 ± 3.75	3.11 ± 0.51	7.14 ± 1.39	3.94 ± 1.27	8.37 ± 0.01
21.70	1485	Deydro-β-ionone	N	-	-	5.30 ± 0.50	-	1.17 ± 0.36
21.80	1486	β-Ionone	N	4.24 ± 0.05	3.08 ± 0.11	0.71 ± 0.23	2.54 ± 0.38	5.84 ± 1.19
21.99	1499	Germacrene D	S	-	0.99 ± 0.02	-	-	-
22.70	1530	δ-Cadinene	S	2.03 ± 0.24	2.72 ± 0.22	-	2.25 ± 0.28	-
22.80	1538	Dihydroactinidiolide	OM	0.70 ± 0.10	-	-	-	-
22.90	1545	Eudesma-3,7(11-diene)	S	0.38 ± 0.07	-	-	-	-
23.20	1554	Nerolidol oxygenated	S	-	-	-	3.55 ± 0.76	-
23.20	1554	Nerolidol oxygenated	S	-	-	-	3.55 ± 0.76	-
24.00	1582	Spathulenol	OS	11.78 ± 1.02	30.87 ± 0.15	8.53 ± 2.34	13.98 ± 1.39	25.86 ± 1.76
24.10	1585	Caryophyllene oxide	OS	15.80 ± 0.42	14.32 ± 0.66	2.76 ± 2.58	17.46 ± 1.48	14.11 ± 0.28
24.40	1598	Ledol	OS	4.05 ± 0.21	-	-	-	-
24.50	1603	Globulol	OS	1.47 ± 0.04	-	-	-	-
24.70	1607	Humulene epoxide II	OS	14.15 ± 0.78	-	1.58 ± 0.14	5.71 ± 0.14	7.08 ± 006
25.20	1631	1,7,7-Trimethyl-2-vinylbicyclo [2.2.1]hept-2-ene (Vinylbornene)	-	5.21 ± 0.27	-	-	-	-
25.40	1634	Longipinocarveol	OS	1.68 ± 0.03	-	-	2.57 ± 0.26	-
25.50	1647	τ-Muurolol	OS	1.75 ± 0.50	-	-	-	-
25.70	1659	α-Cadinol	OS	5.04 ± 0.50	11.99 ± 0.39	-	4.36 ± 0.01	6.32 ± 0.44
27.70	1745	Octanal 2-phenylmethylene	A	-	-	-	0.85 ± 0.27	0.31 ± 0.16
27.90	1768	Tetradecanoic acid	CA	-	0.36 ± 0.25	1.87 ± 0.93	1.58 ± 1.17	0.54 ± 0.63
28.30	1785	Anthracene	H	-	-	-	-	0.68 ± 0.05
28.70	1800	Octadecane	H	-	-	1.04 ± 0.38	-	-
29.60	1850	4,8,12-Tetradecatrienal-5,9,13-trimethyl	A	-	-	1.91 ± 0.59	-	1.08 ± 0.05
30.40	1880	1-Hexadecanol	AL	-	0.63 ± 0.01	3.25 ± 1.85	1.49 ± 1.57	1.87 ± 1.02
34.20	1881	Cyclohexadecane	H	-	1.39 ± 0.00	-	0.99 ± 0.54	-
34.50	1900	Nonadecane	H	-	-	0.81 ± 0.24	-	-
34.80	1909	Methyl hexadecanoate	E	-	-	1.89 ± 0.47	-	-
35.00	1922	Dibutyl phtalate	E	-	-	9.24 ± 3.89	-	-
35.90	2108	Bisphenol A	PH	-	0.10 ± 0.06	3.82 ± 0.48	2.16 ± 0.30	-
39.60	2360	2-Methyltricosane	H	-	-	1.23 ± 1.15	-	-

^(a)^ Linear Retention Index (LRI) calculated for all components using a homologous series of *n*-alkanes analyzed under the same conditions as the samples; (-) not detected. A—aldehyde, AL—alcohol, PP—phenylpropanoid, S—sesquiterpene, N—norisoprenoid, K—ketone, OM—oxygenated monoterpene, OS—oxygenated sesquiterpene, CA—carboxylic acid, HC—hydrocarbon, E—ester, and PH—phenol. BSB3 = botanically identified sample, batch 3; CS1B2 = commercial sample brand 1, batch 2; CS2B1 = commercial sample brand 2, batch 1; CS3B2 = commercial sample brand 3, batch 2; CS4B2 = commercial sample brand 4, batch 2. Relative percentage as the mean ± standard deviation (duplicate).

## Data Availability

Data is contained within this article.

## Sample Availability

Samples of the compounds are available from the authors.
